# Polysaccharides Used in Biosorbents Preparation for Organic Dyes Retaining from Aqueous Media

**DOI:** 10.3390/polym14030588

**Published:** 2022-01-31

**Authors:** Daniela Suteu, Alexandra Cristina Blaga, Carmen Zaharia, Ramona Cimpoesu, Adrian Cătălin Puițel, Ramona-Elena Tataru-Farmus, Alexandra Maria Tanasă

**Affiliations:** 1 Department of Organic, Biochemical and Food Engineering, “Cristofor Simionescu” Faculty of Chemical Engineering and Environmental Protection, ”Gheorghe Asachi” Technical University of Iasi, D. Mangeron Blvd., No. 73A, 700050 Iasi, Romania; danasuteu67@yahoo.com (D.S.); acblaga@tuiasi.ro (A.C.B.); alexandra_tanasa20@yahoo.com (A.M.T.); 2 Department of Environmental Engineering and Management, “Cristofor Simionescu” Faculty of Chemical Engineering and Environmental Protection, ”Gheorghe Asachi” Technical University of Iasi, D. Mangeron Blvd., No. 73A, 700050 Iasi, Romania; 3 Department of Materials Science, “Cristofor Simionescu” Faculty of Materials Science and Engineering, ”Gheorghe Asachi” Technical University of Iasi, D. Mangeron Blvd., No. 73A, 700050 Iasi, Romania; ramona.cimpoesu@academic.tuiasi.ro; 4 Department of Natural and Synthetic Polymers, “Cristofor Simionescu” Faculty of Chemical Engineering and Environmental Protection, ”Gheorghe Asachi” Technical University of Iasi, D. Mangeron Blvd., No. 73A, 700050 Iasi, Romania; 5 Department of Chemical Engineering, “Cristofor Simionescu” Faculty of Chemical Engineering and Environmental Protection, ”Gheorghe Asachi” Technical University of Iasi D. Mangeron Blvd., No. 73A, 700050 Iasi, Romania; ramona-elena.tataru-farmus@academic.tuiasi.ro

**Keywords:** biosorption, polysaccharides as biosorbent, organic dye, *Saccharomyces pastorianus* immobilized, sodium alginate

## Abstract

Natural polymers can themselves be efficient as materials with biosorptive properties but can also be used to transform microbial biomass into an easy-to-handle form, respectively, into biosorbents, through immobilization. The article aims to study biosorbents based on residual microbial biomass (*Saccharomyces pastorianus* yeast, separated after the brewing process by centrifugation and dried at 80 °C) immobilized in sodium alginate. The biosorptive properties of this type of biosorbent (spherical particles 2 and 4 mm in diameter) were studied for removal of reactive dye Brilliant Red HE-3B (with concentration in range of 16.88–174.08 mg/L) from aqueous media. The paper aims at three aspects: (i) the physico-chemical characterization of the biosorbent (Scanning Electron Microscopy (SEM), Energy-dispersive X-ray spectroscopy (EDX) and Fourier Transform Infrared (FTIR) spectra); (ii) the modeling of biosorption data in order to calculate the quantitative characteristic parameters using three equilibrium isotherms (Langmuir, Freundlich, and Dubinin–Radushkevich—DR); and (iii) the evaluation of thermal effect and the possible mechanism of action. The results of the study showed that biosorption capacity evaluated by Langmuir (I) model is 222.22 mg/g (ϕ = 2 mm) and 151.51 mg/g (ϕ = 4 mm) at 30 °C, and the free energy of biosorption (E) is in the range of 8.45–13.608 KJ/mol (from the DR equation). The values of thermodynamic parameters suggested an exothermic process due the negative value of free Gibbs energy (ΔG^0^ = −9.031 kJ/mol till −3.776 kJ/mol) and enthalpy (about ΔH^0^ = −87.795 KJ/mol). The obtained results underline our finding that the immobilization in sodium alginate of the residual microbial biomass of *Saccharomyces pastorianus* led to an efficient biosorbent useful in static operating system in the case of effluents with moderate concentrations of organic dyes.

## 1. Introduction

Water pollution is considered one of the most important environmental problems due to its essential role in life (metabolism, irrigation, industrial) since the presence of pollutants is a severe threat to ecosystems and public health [1]. 

Microbial cells can be used for the removal of pollutants from aqueous effluents through biosorption, a passive process that implies inactive biomass able to adsorb toxic substances on its surface [1,2]. Nonliving biomass of bacteria, fungi, or algae are potential biosorbents, as they are relatively abundant and inexpensive (results as by-product in industrial fermentation) [3,4,5]. Advantages of using inactive biomass include having no nutrients or energy requirements, better sorption capacity, rapid processes, and no toxic effects of the contaminants on the cells. Even in inactive form (nonliving), microorganisms have the capacity to bind different molecules, but their use in free form is limited due to difficult separation (small size, less operational stability, difficult recovery and reusability) [3]. Immobilization of microorganisms can overcome these downsides. By immobilization through different techniques using natural polysaccharides, including alginate, chitosan, starch, and cellulose, better-suited biosorbents (easier to handle, reusable, more stable and robust) can be obtained [4,6]. 

Polysaccharides have multiple uses, namely as drugs, as bonding agents, and as drug carriers, and because they are abundantly present in the environment, the techniques for their processing are very well known and can be functionalized by different techniques (synthetic and protein-related techniques) [7]. Recently, polysaccharides have also been proposed as promising biomolecules used for the production of biosorbents based on residual biomass [8], as they are found in almost all living organisms, such as seaweeds (alginate, agar, carrageenan), plants (cellulose, hemicelluloses, pectin), microorganisms (dextran and xanthan gum), and animals (chitin) [9]. 

Microbial-based biosorbents possess the ability to retain pollutants from wastewater due to a specific surface area and porosity but mostly to a high content of polysaccharides (cellulose, hemicellulose, lignin, pectin, chitin), which contain numerous functional groups (carboxyl, hydroxyl, and carbonyl groups) capable of binding different compounds by forming chemical complexes or/and numerous ion exchanges [10]. 

The types and the number of pollutants that are being removed through biosorption are consistently expanding, including metals, dyes, antibiotics and other pharmaceuticals, and persistent organic pollutants (Lindane, 2,4-Dichlorophenol, 2-Chlorobiphenyl, Pentachloro-nitrobenzene) [1,2,3,4,5]. Biosorption using microbial biomass processes is influenced by several physiochemical parameters, such as pH, temperature, biomass dosage, initial pollutant concentration, contact time, type of pollutant, and type of biosorbent [11].

Dye retention from wastewaters is extremely important for the ecosystem; thus, developing efficient and cost-effective removal techniques is a permanent concern/threat. The current methods include the adsorption of dye to mineral- or carbon-based matrix [12,13], oxidation processes [14], coagulation [15], reverse osmosis [16], and membrane filtration [17], but they have several constraints regarding cost, feasibility, environmental impact, efficiency, operational difficulty, toxic by-product handling, etc. Biosorption can be a sustainable wastewater treatment as an environmentally friendly and affordable treatment for removing dyes from water bodies [6,18].

The use of yeast (unicellular fungi) as a biosorbent offers more advantages: large available quantities, as it is used in large-scale cultivation (brewing, winery); low safety risks; and ease of use. The structure of the fungal cell wall includes mannan, glucan, and galactan in its outer layer and chitin, cellulose, or non-cellulose glucan in its inner layer. *Saccharomyces cerevisiae* (*S. cerevisiae*) has a composition based on the manan–glucan complex and a chitin content of only 1% [1]. *Saccharomyces pastorianus*, a lager brewing strain, has a more hydrophilic surface than *S. cerevisiae* and is deposited at the end of the fermentation on the bottom of the bioreactor. Its cell wall consists of insoluble glycogen-like polysaccharides that represent 1 to 23% of yeast cell wall dry weight, mainly including mannoproteins and β-glucan, and the wall inner layer is composed of glucans, mainly constituted by (β1 → 3)-Glucose residues (85%), (β1 → 6)-Glucose residues (15%), and (α1 → 4)-Glucose residues with (α1 → 4,6)-Glucose covalently linked to (β1 → 6)-glucans [19]. 

Several studies analyzed the use of yeasts for dye retention, obtaining promising results; *S. cerevisiae* retained Brilliant Red HE-3B with a maximum biosorption capacity of 104.167 mg/g in free form [20]. For the removal of Reactive Red 239 (RR239), Reactive Black B (RBB) and Direct Blue 85 (DB85) maximum biosorption capacities of 152.9, 162.7, and 139.2 mg/g were obtained using waste beer yeast slurry [21]. Remazol Red RB was retained from wastewater using *S. cerevisiae* with a maximum adsorption capacity of 0.07 mg/g at pH 6 [22]. Baker’s yeast (*S. cerevisiae*)-MnO_2_ composites, produced by direct oxidation of yeast with KMnO_4_ under acidic conditions, were used as biosorbent to remove the triphenylmethane dye, Malachite green (MG), from an aqueous solution obtaining maximum biosorption capacity of 243.9 mg/g [23]. *Saccharomyces cerevisiae* immobilized on the surface of Fe_3_O_4_ magnetic nanoparticles was used as biosorbent for the removal of methyl orange from aqueous solution in a batch system, obtaining 277.1482 mg/g sorption capacity [24]. 

Sodium alginate is a bio-polysaccharide found in various marine organisms (especially brown algae) that include mannuronic acid (1,4-linked hexuronic acid or β-d-mannuronopyranosyl residues) and guluronic acid (α-1-guluronopyranosyl residues) in different ratios and sequences, characterized by high availability, biodegradability, low cost, long-term polymer stability, and biocompatibility [25,26,27]. 

The aim of this paper is to investigate the biosorptive behavior of a new type of biosorbent based on residual microbial biomass of *Saccharomyces pastorianus* immobilized in sodium alginate. In this purpose, the biosorption balance will be studied by modeling the experimental data using three of the most applied adsorption isotherms and by thermodynamic and kinetic study. The adsorbent material is physico-chemically characterized before and after the biosorption process of the reactive dye, Brilliant Red HE-3B. 

## 2. Materials and Methods

### 2.1. Materials

Biomass. The yeasts of *Saccharomyces pastorianus (Saccharomycetaceae* family, hybrid between *Saccharomyces cerevisiae* and *Saccharomyces eubayanus* [10]) are by-products in the brewing processes and therefore are available in large quantities in the form of residual yeast. At the end of the brewing process (Albrau, Onești, Romania), the *Saccharomyces pastorianus* residual biomass was separated by centrifugation (8000 rpm), dried at 80 °C, and then immobilized in sodium alginate.

Biosorbent. The biosorbent used in the experimental biosorption studies was based on the immobilization of residual biomass (*Saccharomyces pastorianus*) on sodium alginate. For immobilization, a 1% sodium alginate solution prepared in distilled water at 70 °C was mixed with 5% residual biomass and dripped after complete homogenization in 1% calcium chloride solution (prepared in distilled water at 5 °C) by two different capillaries, thus obtaining spherical particles with diameters of 2 mm (ϕ1) and 4 mm (ϕ2).

**Figure 1:**
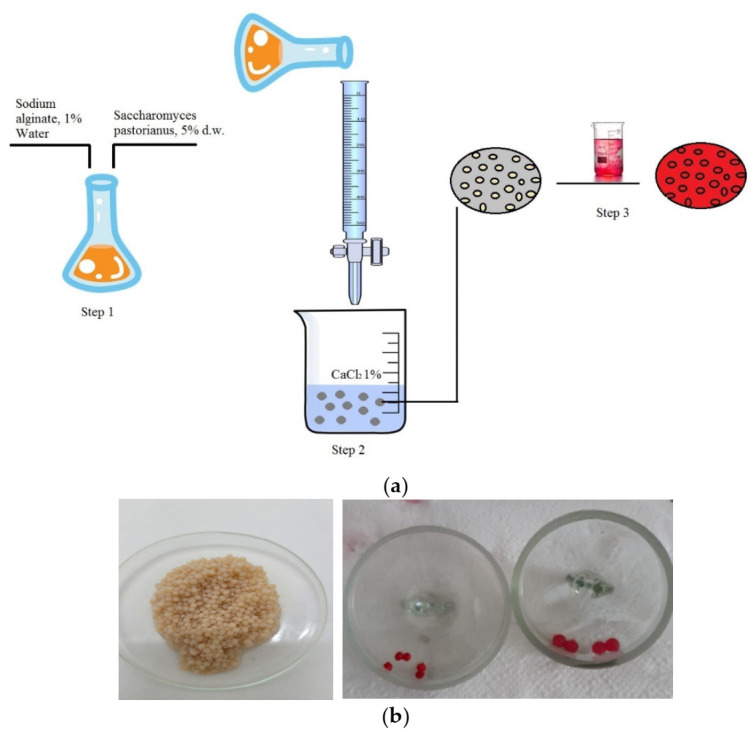
(**a**) Schematic representation of the immobilized and biosorption processes; (**b**) immobilized biomass granules before and after dye biosorption.

The schematic representation of all steps involved in the preparation of biosorbent with *Saccharomyces pastorianus* is shown in Figure 1a. All granules of polymeric consortium, residual biomass-sodium alginate, have uniform size (Figure 1b) and are stable during storage in aqueous solution of calcium chloride 10 mM at a temperature of 5 °C, without manifested adhesion phenomena.

The prepared biosorbents with spheric shape and mesoporous surface possess multiple sorption sites. They have a large surface area with several functional groups available from microbial cells (amine, carboxyl, phosphate, etc.). Amine groups especially are able to bind organic compounds through electrostatic interactions, as shown by Vijayaraghavan and Yun [28]. *Saccharomyces pastorianus* cell wall contains mannoproteins, and N-linked to the nitrogen of asparagine or arginine type mannoproteins is the majority [29]. The drying treatment can increase the cell porosity by disrupting different bonds [30] making available more adsorption sites and improving the biosorption efficiency.

**Figure 2:**
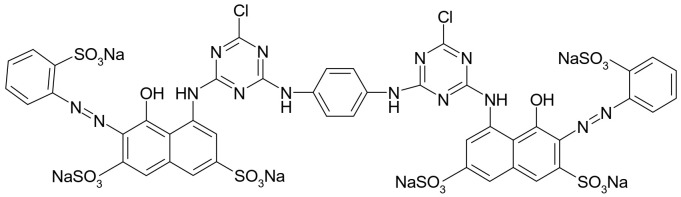
Chemical structure of reactive dye, Brilliant Red HE-3B—C.I. 25810.

Adsorbate. A reactive dye, Brilliant Red HE-3B (BRed) (MW = 1430 g/mol, λmax = 530 nm, from Bezema Colour Solutions, Montlingen, Switzerland), with chemical structure showed in Figure 2, was selected as chemical pollutant (reference model of reactive dye) of aqueous system for this study. The chosen dye is part of the class of reactive dyes frequently used for dyeing natural cotton fibers, which makes it, along with its structure, suitable to be considered as a study model in this situation.

The stock solution with a concentration of 500 mg dye/L was prepared from the commercial form of the dye powder. From this, the working solutions were prepared by appropriate dilution with distilled water.

### 2.2. Methods 

#### 2.2.1. Batch Biosorption Methodology

Experimental biosorption studies were performed by contacting, in 50-mL Erlenmeyer flasks, different amounts of biosorbent with 5% dry matter (d.w.), pre-washed with distilled water to remove traces of calcium chloride solution, which could cause precipitation of the dye, with 25 ml of reactive dye solution in different initial concentrations (in the range of 16.88–174.08 mg/L) (Figure 1a). The pH values were adjusted with 1N HCl and/or 1N NaOH solution and the set temperature (5 °C, 30 °C, 45 °C) was kept constant using a cooler (for 5 °C) and a thermostatic oven Poleko SLW53 model (Pol-Eko-Aparatura sp.j., Wodzisław Śląski, Poland) (for 30 °C, 45 °C). The contact time of the solid-aqueous phases was about 24 h. The dye content of the equilibrium solution was determined spectrophotometrically using a Shimadzu UV-1280 UV-VIS spectrophotometer (Shimadzu Corporation, Kyoto, Japan) at a maximum dye wavelength of 530 nm, in compliance with Lambert–Beer law. To obtain the biosorption capacity characteristic to the tested biosorbent (q, mg of dye/g of biosorbent), Equation (1) was used:

(1) $$q=\frac{C_{0}-C}{G}\cdot V$$

where C_0_ and C are the dye initial and the equilibrium (residual) concentration in solution (mg/L), G is the amount of biosorbent (dry matter (d.w.) from alginate granule) (g), and V is the volume of solution (L).

#### 2.2.2. Physicochemical Characterization of Biosorbent

The characterization of the biosorbent obtained by immobilizing the residual biomass of *Saccharomyces pastorianus* in sodium alginate was done before and after the dye-retention process to identify potential changes in its structure due to the dye retention. In this regard, a number of advanced analysis methods have been used (SEM, FTIR) to highlight the internal structure and a number of functional groups that could be responsible for the biosorbent properties.

Lyophilization. Biosorbent characterization studies based on residual microbial biomass immobilized in sodium alginate, having diameter ϕ2 = 4 mm, were performed on freeze-dried samples. The operation was performed using a Labconco lyophilizer (Labconco, Kansas City, MO, USA) and working with the following process parameters: 0.05 mBar, −50 °C, time—6h. 

Scanning Electron Microscopy (SEM) was carried out to characterize the surface micromorphology of the biosorbent based on *Saccharomyces pastorianus* immobilized in sodium alginate before and after the biosorption process. A scanning electron microscope, VegaTescan LMH II (Tescan Orsay Holding, Brno-Kohoutovice, Czech Republic), detector SE, WD 15.5 mm, 30 kV, HV, VegaTC software with EDS detector XFlash 6/10 Bruker, Automatic mode and Mapping distribution of elements, Esprit 2.2 software was used.

The Fourier Transform Infrared (FT-IR) spectroscopy was used to identify the functional groups existing in the original biosorbent that could be involved in the binding of dye molecules. In addition, FTIR spectra were drawn for the biosorbent after the biosorption process of the dye to highlight precisely how the molecules bind to the biosorbent but also the possible changes in the initial structure of the biosorbent as a result of the biosorption process. For this purpose, the FTIR spectra were recorded using a Bruker Vertex 70 FT-IT spectrophotometer (Bruker, Karlsruhe, Germany) in total attenuated reflectance mode in the wavelength range 4000–400 cm^−1^ with a resolution of 2 cm^−1^ and 32 acquisitions, at room temperature.

#### 2.2.3. Modeling the Biosorption Experimental Data

For experimental data modeling, three biosorption equilibrium models, Freundlich (F) [31], Langmuir (L) [32], and Dubinin–Radushkevich (DR) [33], synthesized in Table 1, were applied, and comparison with other findings reported by different researchers in Table 2 was achieved.

### 2.3. Thermodynamic Parameters of the Biosorption Process

Based on the value of the Langmuir constant, K_L_ at three temperatures and three thermodynamic parameters were determined using the equations introduced in Table 3 [34,35].

### 2.4. Kinetic Study of the Biosorption Process

The influence of contact time of dye and biosorbent on the biosorbent itself, based on the residual biomass of *Saccharomyces pastorianus* immobilized in sodium alginate, was studied in batch experiments, mixing 0.271 g (with 5% d.w.) of biosorbent and 100 mL of dye solution (19.2 mg/L, pH = 2) at temperature of 20 °C for time intervals ranging from 10 min to 24 h. At the end of the biosorption process, the biosorbent was separated by filtration, and the dye content of the filtrate (remaining aqueous phase) was determined spectrophotometrically. Using Equation (1), the biosorption capacity q_t_ and q were calculated. The extent of biosorption was expressed by the fractional attainment of equilibrium, F (Equation (2).

(2) $$F=\frac{q_{t}}{q}$$

where q_t_ and q (mg/g) are the dye biosorbed at time *t* and at equilibrium (24 h).

### 2.5. Error Analysis

Even if the linear regression coefficient, R^2^, is the most commonly used error function in many studies, in the literature, there are other complex analysis methods for error, such as average relative error, a hybrid error function, Marquardt’s percent standard deviation, nonlinear chi-square test, the sum of squares of the errors, standard deviation of relative errors, sum of the absolute errors, and Spearman’s correlation coefficient, which have been used to establish the most proper equation [36,37,38]. The chi-square test, χ^2^, and the residual root mean square errors, RMSE, were selected to be used in this study. The standard equations are as follows:

(3) $$\chi^{2}=\sum_{i=1}^{N}\frac{{\left(q_{e,\exp}-q_{e,calc}\right)}^{2}}{q_{e,calc}}$$

(4) $$RMSE=\sqrt{\frac{1}{N-2}\sum_{i=1}^{N}\frac{{\left(q_{e,\exp}-q_{e,calc}\right)}^{2}}{q_{e,calc}}}$$

where q_e,exp_ and q_e,calc_ represent the experimental and calculated values of sorption capacity (mg/g), and N is the number of experimental data.

## 3. Results and Discussion

### 3.1. Analysis of the Biosorbent Using SEM, EDAX, and FT-IR Spectra

**Figure 3:**
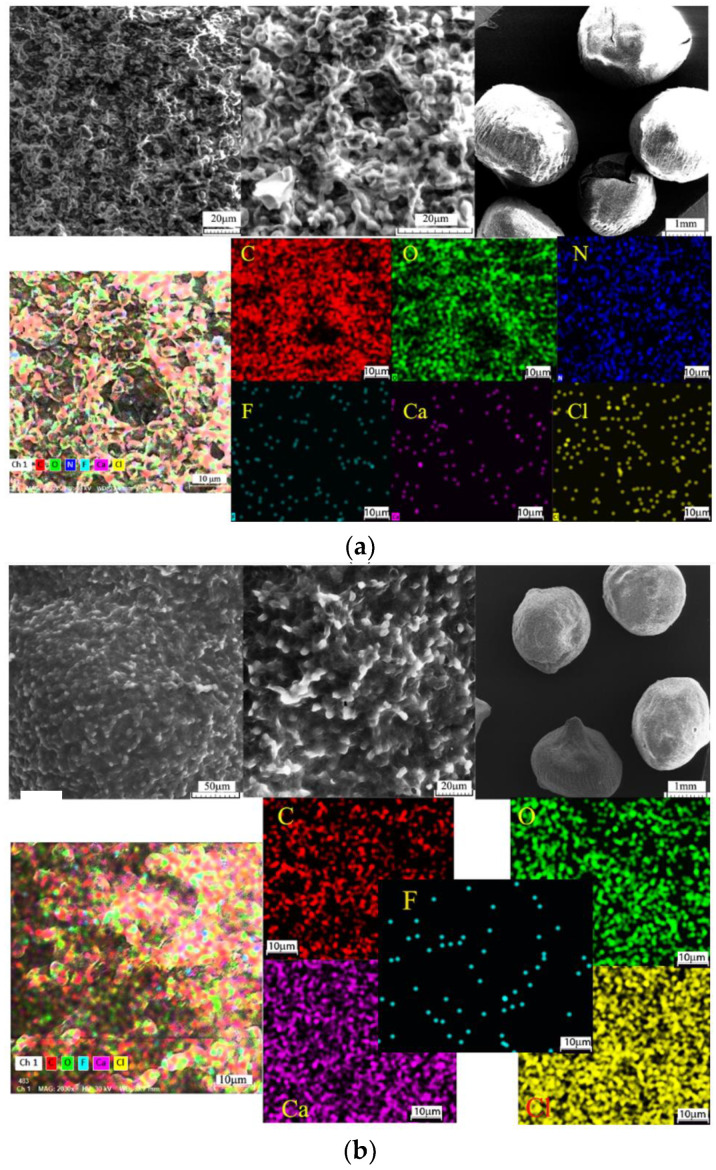
Scanning electron microscopy (SEM) and mapping of C, O, N, F, Ca, and Cl elements of the biosorbent after (**a**) and before (**b**) Brilliant Red HE-3B dye biosorption.

Scanning electron microscopy was used to study the biosorbent (based on the residual biomass of *Saccharomyces pastorianus* immobilized in sodium alginate, particle with ϕ 2 = 4 mm) morphology and also certain information about pores distribution and location. Images obtained at 20×, 500×, and 1000× magnifications are shown in Figure 3.

The SEM imagines for biosorbent illustrate the presence of mesoporous structure (50 μm and 20 μm) in the analyzed material, which is slightly more uniform after the biosorption process of Brilliant Red HE-3B dye on it. The surface variation profile presents a mean value of 121 μm for the initial surface and 118 μm after the biosorption process.

The particles, shown in Figure 3a,b, have a regular geometric shape with similar dimensions (average radius: 1.12 ± 0.04 mm before Brilliant red HE 3B dye absorption and 1.26 ± 0.17 mm after). The adjacent peaks presented on initial particles, shown in Figure 3 b, disappear after dye biosorption. Pores present an average value of radius of 3.19 μm with a standard deviation of 0.63 and after biosorption of 3.06 ± 0.39 μm, presenting a small reduction. All chemical elements present a homogeneous spread before and after Brilliant Red HE 3B dye biosorption.

**Figure 4:**
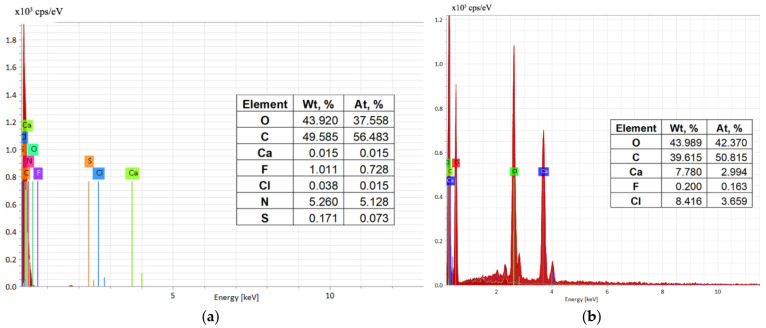
Energy-dispersive X ray (EDAX) spectra of the biosorbent after (**a**) and before (**b**) Brilliant Red HE-3B dye biosorption.

Energy-dispersive X-ray spectroscopy (EDS, EDAX, EDX, EDXS, or XEDS technique) was used for the elemental analysis of biosorbent samples before and after the biosorption of Brilliant HE-3B Red Dye in order to evaluate the process performance (Figure 4).

The EDAX spectrum (Figure 4a,b) illustrates the presence on the surface of various elements originated from microbial biomass and sodium alginate and also retained dye, the increase of carbon, and appearance of nitrogen and sulfur atoms, respectively. All changes after the biosorption suggest the reactive dye was retained on the surface of the biosorbent (polymeric composite material) (Figure 4a). A certain difference is observed in the amounts of calcium before and after biosorption, which can be due to the granules washing operation with distilled water (practiced before use in the biosorption process).

**Figure 5:**
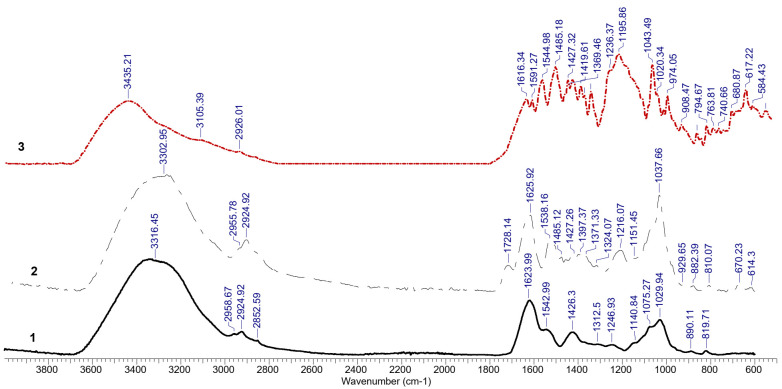
FT-IR spectra of the biosorbent before (1) and after (2) biosorption and of Brilliant Red HE-3B dye (3).

The FTIR spectra obtained for the biosorbent before and after the dye biosorption process as well as the spectrum of the studied dye Brilliant Red HE-3B are presented in Figure 5.

The analysis of the spectra presented in Figure 5 leads to a first important finding, namely the appearance of new peaks in the structure of biosorbent loaded with dye (2) compared to the initial structure of biomass (1) and decreased intensity of possible peaks due to biosorption process. Starting from the relevant peaks observed in the structure of the dye (3) and from those of the biomass (1), some observations can be made regarding the spectrum of the biomass loaded with dye (2) that support the idea of dye retention on this type of biosorbent. Regarding the dye FTIR spectrum (3), the absorption bands were assigned as follows: the peak at 3435 cm^−1^ can be seen in the pure dye FTIR spectrum (3) and was assigned N-H in amine groups since no OH are present in its structure. In case of the biosorbent FTIR spectra (1), the peak at ~3300 cm^−1^ was assigned to the O-H stretching vibration, hydroxyl groups from biosorbent and water molecules, taking into account that the adsorbent is mainly composed of polysaccharides; the shoulders appearing at 2920 and 2820 cm^−1^ to the antisymmetric stretching vibrations of C–H bonds; furthermore, the 1616 cm^−1^ band was assigned to C=N double bond stretching vibrations, the aromatic ring stretching was noticed at 1590 cm^−1^, while the 1545 cm^−1^ band was assigned to the N–H deformation vibrations. The N=N stretching vibration was assigned at 1485 cm^−1^. The O=S=O in the sulfonic groups showed a sharp peak at 1323 cm^−1^ as a result of antisymmetric stretching vibrations, while the 1165 cm^−1^ band corresponding to the group symmetric stretching was overlapped with other functional groups present in the structure of the dye, such as the sulfonic group SO_3_^−^. The peak at about 1040 cm^−1^ was assigned to phenolic hydroxide group present in the structure but also to S–O bond in sulfonic groups.

Concerning the biosorbent (1) and biosorbent after dye biosorption (2) infrared spectra (FT-IR), the following findings could be observed: the broad OH stretching vibration band was common for both samples. The same statement seems to be true regarding the CH vibration bands occurring at ~2950 and ~2850 cm^−1^. In the case of biosorbent spectrum (1), the band at 1728 cm^−1^ corresponds to C=O stretching occurring in carboxyl and carbonyl groups. This band reduces in intensity in the biosorbent-dye spectrum (2). The band at ~1625 cm^−1^ could be assigned to NH deformation in amine and amino-acids but also in the dye. This band occurs in both spectra and increases in intensity as a result of dye biosorption with possible amine salt formation. The ~1540 cm^−1^ is assigned to secondary amides and occurs as a combination of NH bending and C–N stretching. The band occurring at ~1425 cm^−1^ was assigned to aliphatic hydroxyl group deformation. The band at ~1220 cm^−1^ in the biosorbent spectrum corresponds to the C–N stretching vibrations. Associated with this band and functional group, but of lower intensity, the 1370 cm^−1^ band also occurs. The bands present in 1030 cm^−1^ and ~850 cm^−1^ are assigned to the C–O–C vibrational stretching of ether linkages, both occurring in polysaccharides structure. The O=S=O in the sulfonic groups showed a sharp peak at 1323 cm^−1^ (in dye spectrum-3) and 1312.5 cm^−1^ in biosorbent after biosorption (2) as a result of antisymmetric stretching vibrations, while the 1165 cm^−1^ band corresponding to the group symmetric stretching was overlapped with other functional groups present in the structure of the dye, such as the sulfonic group SO_3_^−^ [39,40] but also to S-O bond in sulfonic groups [41]. These are valuable indicators that suggest the dye retention. Other peaks identified in the biosorbent spectrum after biosorption that confirm the dye retention process are 1728.14 and 1538.16 cm^−1^ assigned to C=O; 1625.92 cm^−1^ assigned to aromatic ring stretching; 1485.12 cm^−1^ assigned to C=O; and 1216.07 cm^−1^ assigned to COC.

### 3.2. The Value of Point of Zero Charge (pH_PZC_) for Biosorbent

One parameter by which the electric charge of the surface of an adsorbent material can be assessed is the zero-charge point (pH_PZC_). Depending on its value, the behavior of the material in the interaction with the chemical species that must be retained can be specified. This parameter was determined for the biosorbent based on residual biomass immobilized in sodium alginate in our previous study [8] and is of 5.4. Thus, depending on this value, it can consider that for pH values < pH_PZC_, the biosorbent surface will be positively charged (due to the characteristic groups that will be ionized) and will be able to react with anionic species through electrostatic interactions and hydrogen bonds. In the case of pH values > pH_PZC_, the surface of the biosorbent becomes negatively charged (due to the dissociation of characteristic functional groups), which makes it suitable for cation exchanges and/or electrostatic interactions with cationic species.

### 3.3. Modeling the Biosorption Equilibrium Process

**Figure 6:**
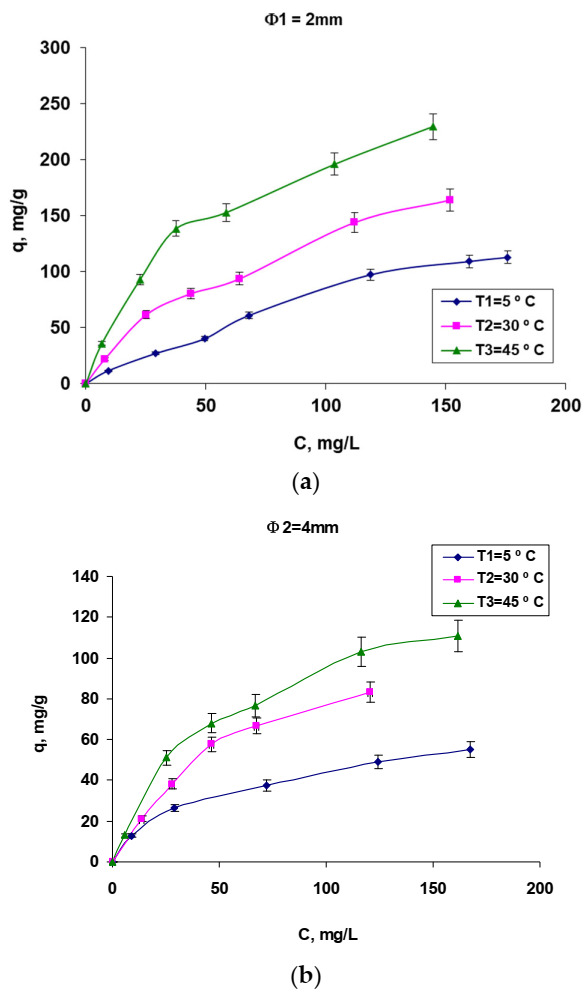
Biosorption isotherms of Brilliant Red HE-3B reactive dye on biosorbent based on residual *Saccharomyces pastorianus* biomass immobilized in sodium alginate, in the form of two-dimensional granule: ϕ 1 = 2 mm (**a**) and ϕ 2 = 4 mm (**b**). Conditions: pH = 3, contact time = 24 h, amount of biosorbent = 2.8 g/L (in the case of ϕ 1) and 2.4 g/L (in the case of ϕ 2) (5% d.w.).

Taking as a starting point the findings from a previous study regarding the operating factors that influence the biosorption process of Brilliant Red-HEB 3B reactive dye (pH = 3, contact time of 24 h, temperature of 25 °C and the concentration of biosorbent in range of 2.4 and 2.8 g/L (with 5% d.w.) depending on the diameter of the biomass-based granules [42]), in this study, we proceed to the analysis of the biosorption balance for Brilliant Red HE-3B reactive dye using a biosorbent obtained by immobilization of residual biomass *Saccharomyces pastorianus* in sodium alginate. In order to achieve this objective, the quantitative characteristic parameters that describe the process and the thermodynamic and kinetic parameters were determined, and the evaluation of the biosorption mechanism was initiated. The experimental data (made in duplicate) led to the biosorption isotherms shown in Figure 6. These were processed using three isotherm models from the scientific literature (Table 1), which allowed, by graphical representation of their linearized equations, the determination of quantitative characteristic parameters (Table 1). 

The isotherms shown in Figure 6a,b illustrate that the retention of the Brilliant Red HE-3B dye on the tested biosorbent strongly depends on the temperature and the size of the granules. Thus, it is observed that the retention of the dye is positively influenced by a small granule diameter (ϕ 1, Figure 6a) because it ensures a much larger contact surface between the dye molecules from solution and the biosorbent. In addition, the increase in temperature positively influences the biosorption regardless of the size of the biosorbent granules, but it is not possible to work above values of 45–50 °C because the biosorbent denaturation occurs. The alignment of the curves indicates a type of “L” isotherm, subgroup 2 according to Gills’ classifications [43], these being in fact the classic Langmuir isotherm that is based on the surface biosorption of vertically oriented molecules by particularly strong intermolecular bonds.

Analyzing the systematized data in Table 1, we can draw some conclusions regarding the studied biosorption process. It is obvious that the biosorption of Red Brilliant HE-3B dye on the tested biosorbent (biosorption capacity value) is more efficient for smaller-diameter granules regardless of each tested temperature. This is because in this case, a larger contact surface, a better contact between the dye molecules, and the immobilized biomass are ensured, which is favorable for the diffusion process. There is also a positive influence of temperature rise, suggesting an exothermic process. By comparatively analyzing the values of the regression coefficients, R^2^, the experimental data correspond best to the Langmuir 1 model for all three studied temperatures.

The model, through the value of the biosorption energy (E), allows a series of preliminary findings regarding the mechanism of the biosorption process (that can be physical or chemical) from the information provided by the value of the biosorption energy (E). The values of E obtained in the range of 8.28–11.18 kJ/mol characterize the biosorption processes based on physical bonds, such as van der Waals interactions, hydrogen, dipole-dipole interactions, and electrostatic attraction established between the positively charged surface of the biosorbent and the functional groups of dye [44].

**Figure 7:**
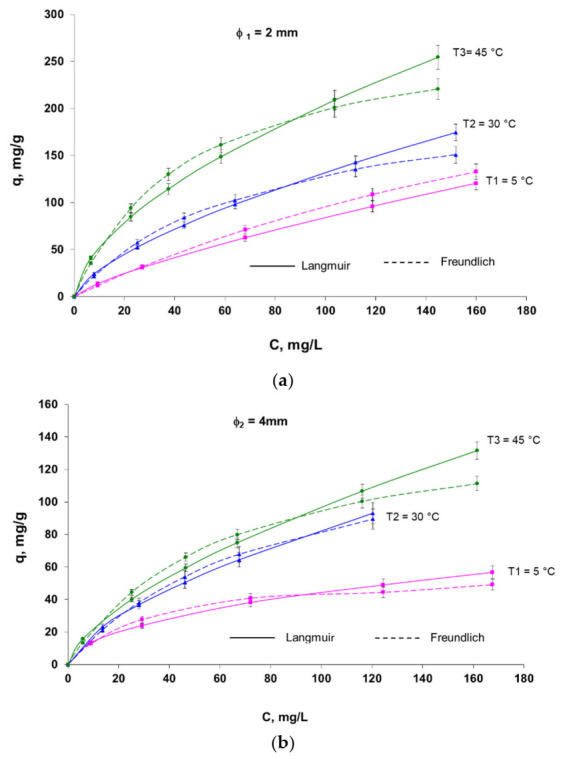
The biosorption isotherms (Langmuir I and Freundlich) of reactive Brilliant Red HE-3B dye onto immobilized residual microbial biomass-based biosorbent. Conditions: ϕ1 = 2 mm (**a**) and ϕ 2 = 4 mm (**b**), pH = 3, contact time = 24 h, amount of biosorbent = 2.8 g/L (in case of ϕ 1) and 2.4 g/L (in the case of ϕ 2) (with 5% d.w.).

The isotherms obtained based on the biosorption data resulting from the proposed isothermal models (especially Langmuir and Freundlich) (Table 1) of the Brilliant Red HE-3B reactive dye on the biosorbent based on the *Saccharomyces pastorianus* residual biomass immobilized in sodium alginate are shown in Figure 7.

The value obtained for the biosorption capacity (Langmuir I model) for 30 °C was compared with other values of the biosorption capacity existing in the literature for other different types of microbial biomass in free form or immobilized on sodium alginate matrices to remove organic dyes from aqueous solutions (Table 2). The results obtained in this study are comparable with literature results for yeast retention of dyes, with higher values being obtained for immobilized biomass compared to the free form.

### 3.4. Analysis of the Proposed Thermodynamic Parameters

Three main thermodynamic parameters were calculated (Table 3) [34,35,53]. The calculation for the thermodynamic parameters was done in the case of granules with diameter ϕ 1 = 2 mm, in which the best regression coefficients and the highest values of biosorption capacity were obtained. For this purpose, the Langmuir constant (K_L_, L/g) obtained in the case of granules with this diameter was used in all relations from Table 3. 

The negative values obtained for ΔG^0^ suggest that, in general, the biosorption of the reactive dye Red Brilliant HE-3B on the tested biosorbent, based on residual bacterial biomass of *Saccharomyces pastorianus* immobilized in sodium alginate, could be considered a spontaneous process. Because the values of ΔG^0^ are between −20 and 0 kJ/mol, it may be an indication that the biosorption process is based on a physical mechanism. This observation confirms the finding offered by the DR model according to which the values of the average free energy of adsorption (E) are in the field of physical adsorption. 

The value for ΔH^0^ is positive, which proves the use of energy to increase the degree of biosorption, which indicates an endothermic biosorption process positively influenced by the increase in temperature. The positive value of ΔS^0^ shows a high affinity of dye molecules for the biosorbent and a random increase at the biosorbent-adsorbed interface during the biosorption phenomenon [45]. In addition, the positive value of ΔS^0^ shows that the total process is based on the dissociative mechanism [45].

### 3.5. Kinetic Studies

#### 3.5.1. Influence of Contact Time

**Figure 8:**
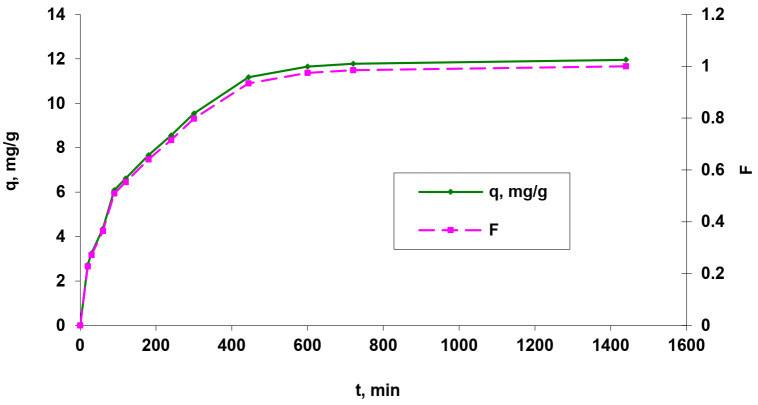
Influence of contact time (q = f(t) and F = f(t)) on reactive dye Brilliant Red HE-3B biosorption onto residual bacterial biomass of *Saccharomyces pastorianus* immobilized in sodium alginate expressed through the amount of dye adsorbed (q) and fractional attainment of equilibrium (F) (biosorbent dose of 2.71 g/L (5% d.w.); pH = 3; temperature of 25 °C).

One of the important operational parameters of a biosorption process is the contact time between the liquid and solid phases. Its influence can be used to determine the time to reach equilibrium by the biosorption system but also to characterize, model, and optimize the process. The effect of the contact time on the Brilliant Red HE-3B reactive dye removal by biosorbent based on residual biomass immobilized in alginate is presented in Figure 8. 

As seen in Figure 8, the values of the fractional attainment of equilibrium rapidly increase with the contact time during the first 500 min, and after that, the rate of dye biosorption became slower, and the time period required for maximum removal of dyes was found to last up to 10 h. Furthermore, the biosorption half-times (t_1/2_) were established to 146 min. In addition, the biosorption capacity (q) has highest value (around 12 mg/g) after at least 480–600 min, which means that very good biosorption efficiency requires high contact times better achieved in a discontinuous treatment regime.

#### 3.5.2. Kinetic Modeling

Three steps are involved in the dyes-adsorption processes [54,55,56]: (i) the diffusion of the dye molecules from bulk solution to the biosorbent surface through the boundary layer (film diffusion); (ii) the diffusion of dye molecules inside of the solid particle (pore diffusion or intraparticle diffusion); and (iii) the interaction of dye with the active sites on the surface of the biosorbent. The biosorption kinetic will be governed by the slowest of one of these steps. 

In general, in the adsorption processes, the most important stage is the diffusion in the liquid film that surrounds the biosorbent particles (external diffusion) and the diffusion in biosorbent particles (internal diffusion).

**Figure 9:**
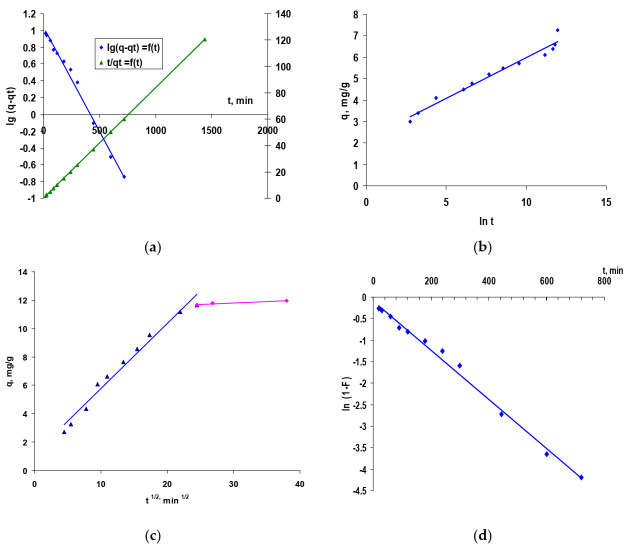
Applicability of pseudo-first order and pseudo-second order diffusion kinetic models (**a**), Elovich model (**b**), intra-particle diffusion model (**c**), and film diffusion model (**d**) to reactive Brilliant Red HE-3B dye biosorption onto residual bacterial biomass of *Saccharomyces pastorianus* immobilized in sodium alginate (biosorbent dose of 2.71 g/L (5% d.w.); pH = 3; temperature of 25 °C).

In order to investigate the mechanism (particle diffusion/film diffusion or chemical reaction) involved in the biosorption of the reactive dye Red Brilliant HE-3B on the studied biosorbent and to establish the decisive step of the biosorption process rate, the kinetic data were processed and interpreted using a series of kinetic models from the literature presented in Table 4. The kinetic parameters related to each model, calculated from the intercepts and slopes of the corresponding linear plots (Figure 9), are presented in Table 4. The assessment of the validation of each model by the experimental data was made based on the value of the correlation coefficients of the linear regression, the R^2^ values.

Comparative analysis of the data summarized in Table 4 and the graphical representations in Figure 9 allowed the formulation of the following findings on the kinetics of the studied biosorption process:

- The graphical representation ln (q − q_t_) versus *t* gives a straight line, which did not pass through the origin of axes. Moreover, the q_e,exp_ values did not agree with the calculated q_e,exp_, with all of these suggesting that the pseudo-first order model is not well fitted for modeling of the kinetic data. The linearity of plots of *t*/q_t_ versus *t*, with a coefficient R^2^ = 1, and the values of q, calculated closer to the experimental value, suggested that the biosorption kinetics onto biosorbent based on residual biomass of *Saccharomyces pastorianus* immobilized in sodium alginate followed a pseudo-second order kinetic model, and the dye-biosorption process could be controlled by chemical biosorption or chemisorption, which involves, for example, valence forces by electron exchange between the two phases involved. Data processing according to the Elovich model achieved values for R^2^ greater than 0.95 in the case of the studied biosorption system (low concentration) and suggests that this model would be appropriate in this case. However, it was observed that the graphical representation q versus ln *t* is linear but does not intersect the origin of the axes, which confirms that chemisorption cannot be the only step that controls the studied biosorption process, and diffusion processes remain a step that could control the Brilliant HE-3B dye biosorption on biosorbent under study.

These three partial findings lead to the conclusion that the rate of biosorption could be governed by either liquid-phase mass transport or intra-particle mass transport. Therefore, in order to obtain more accurate information about the diffusion mechanism, the kinetic data were analyzed using both the intra-particle diffusion model (by Webber–Morris) and the film diffusion model (by McKay) (Figure 9) [55,56].

The graphical representation (Webber–Morris model) in Figure 9c is linear, which induces the idea that intra-particle diffusion takes place, but because neither originates, it does not support the assumption that both diffusion mechanisms (intra-particle and film) are involved in biosorption processes and may be the stage of rate determination. Additionally, the multi-linearity of the graph indicates that there are two or more steps that influence the biosorption process [55,56]. The first part could be regularly associated with film diffusion (external mass transfer) [57], while the second linear part recommends intra-particle diffusion (in the biosorbent structure) [58]. The graphical representation of the McKay model (Figure 9d) confirms that the film diffusion is involved in the biosorption process because the graph ln (1 − F) vs. t is linear and is not, however, the speed limiting step because the line does not pass through the origin.

## 4. Conclusions

The results obtained in this study highlight the fact that biosorbents based on residual microbial biomass immobilized in a polymeric matrix based on polysaccharides (sodium alginate in this case) can be effective biosorbents for retaining organic dyes present in aqueous solutions in moderate concentrations. The biosorption process of the reactive Brilliant Red HE-3B dye on biosorbents with two size particles, obtained by immobilizing the residual biomass of *Saccharomyces pastorianus* in the sodium alginate matrix, was studied. The assessment of biosorbent properties was done by processing experimental data using certain known isotherm models: Langmuir, Freundlich, and Dubinin–Radushkevich, with the Langmuir model being the one that best fits the experimental data in this case.

This study also showed that this biosorption process using a biosorbent based on *Saccharomyces pastorianus* immobilized on sodium alginate for the removal of the Brilliant Red HE-3B reactive dye is more physical than chemical, according to the calculated value of the free biosorption energy (E = 8.28–11.23 kJ/mol, from the DR model equation).

The negative values of the free energy Gibbs (ΔG^0^ = −5.78 and −9.02 kJ/mol) and the positive value of biosorption enthalpy (ΔH^0^ = 16.96 kJ/mol) suggested that the process can be considered spontaneous and endothermic.

## Figures and Tables

**Table 1:** Characteristic parameters for the biosorption of Brilliant Red HE-3B reactive dye onto biosorbent based on residual biomass of *Saccharomyces pastorianus* immobilized in alginate.

Isotherm	Φ1 = 2 mm			Φ 2 = 4 mm		
	5 °C	30 °C	45 °C	5 °C	30 °C	45 °C
**Freundlich**: $q=K_{F}\cdot C_{}^{1/n}$$/\log q=\log K_{F}+\frac{1}{n}\log C_{}$ [31]						
K_F_ ((mg/g) (L/mg)^1/n^)	1.763 ± 0.864	6.131 ± 1.055	13.398 ± 2.924	4.55 ± 0.521	4.395 ± 1.201	5.131 ± 1.445
n	1. 215 ± 0.05	1.4996 ± 0.098	1.4996 ± 0.098	2.0297 ±0.0065	1.569 ± 0.019	1.567 ± 0.019
R^2^	0.9932	0.9834	0.9653	0.9905	0.9646	0.9539
χ^2^	0.00274	0.00488	0.00756	0.00185	0.00465	0.01198
RMSE	0.02615	0.03491	0.04348	0.02486	0.039375	0.06516
**Langmuir**: $q=\frac{K_{L}\;\cdot \;C\;\cdot \;q_{0}}{1\;+\;K_{L}^{}\;\cdot \;C}$ [32]						
**Langmuir I**: $(1/q=f\;(1/C))/\frac{1}{q}=\frac{1}{q_{0}}+\frac{1}{K_{L}\;\cdot \;q_{0}}\;\cdot \;\frac{1}{C}$ [32]						
q_0_ (mg/g)	155.96 ± 47.59	224.47 ± 24.99	295.42 ± 15.753	58.907 ± 4.513	151.15 ± 14.88	153.34 ± 23.58
K_L_ (L/g)	0.0083 ± 0.002	0.0138 ± 1.33 × 10⁻^3^	0.0207 ± 2.32 × 10⁻^5^	0.0303 ± 9.7 × 10⁻^4^	0.012 ± 8.65 × 10⁻^4^	0.0161 ± 1.88 × 10⁻^4^
R^2^	0.9902	0.997	0.9989	0.9942	0.9972	0.9957
χ^2^	0.00199	0.00296	6.578 E-05	0.00579	0.00143	0.00122
RMSE	0.02233	0.00861	0.004055	0.01389	0.00691	0.01744
**Langmuir II**: $(C/q=f\;(C\;))/\frac{C}{q}=\frac{1}{q_{0}\;\cdot \;K_{L}}\;+\;\frac{C}{q_{0}}$ [32]						
q_0_ (mg/g)	303.31 ± 25.79	254.91 ± 26.67	301.75 ± 15.06	68.885 ± 6.398	130.93 ± 8.504	150.151 ± 7.12
K_L_ (L/g)	0.0036 ± 6.89 × 10⁻^4^	0.011 ± 7.69 × 10⁻^5^	0.0198 ± 4.47 × 10⁻^5^	0.0204 ± 0.00203	0.0151 ± 4.686 × 10⁻^6^	0.0176 ± 0.0176
R^2^	0.7614	0.9589	0.9902	0.9752	0.9876	0.9911
χ^2^	0.0523	0.0177	0.00386	0.0604	0.00632	0.0106
RMSE	0.1143	0.0664	0.03107	0.1419	0.0459	0.0516
**Dubinin**–**Radushkevich (DR)**: $q=q_{0}\exp\left(-B\cdot \varepsilon^{2}\right)$/ln q = ln q_0_$\;-\;B\varepsilon 2^{\;}/\varepsilon =\mathrm{RT}\;\ln\left(1+\frac{1}{C}\right)$$/E=\frac{1}{\sqrt{2B}}$ [33]						
q_0_ (mg/g)	2793.92 ± 0.213	2370.86 ± 0.175	2619.4 ± 0.227	13608.95 ± 1.581	219.68 ± 0.549	1506.58 ± 0.286
β (mol^2^ /kJ^2^)	0.0073 ± 3.68 × 10⁻^4^	0.005 ± 2.53 × 10⁻^4^	0.004 ± 2.88 × 10⁻^4^	0.0116 ± 0.0027	0.0024 ± 8.28 × 10⁻^4^	0.0043 ± 3.67 × 10⁻^4^
E (kJ /mol)	8.287 ± 0.2097	9.987 ± 0.131	11.23 ± 0.409	6.563 ± 2.357	14.52 ± 2.76	10.834 ± 0.469
R^2^	0.9899	0.9899	0.9794	0.8569	0.7325	0.9712
χ^2^	1.7786	1.7467	6.2314	10.7264	15.523	5.1719
RMSE	0.6668	0.6608	1.2481	5.979	2.275	1.1371

K_F_ and 1/n, the Freudlich constants associated with the biosorption capacity and intensity (efficiency), respectively; a favorable biosorption corresponds to a value of 1 < n < 10; q_0_ and K_L_, Langmuir constants, q_0_ is the maximum amount of sorbed solute (mg/g), and K_L_ is the constant related to the binding energy of solute (L/mg); q_D_, maximum biosorption capacity (mg/g); β, activity coefficient related to mean biosorption energy; ε, Polanyi potential; E, mean free energy of biosorption (kJ/mol).

**Table 2:** Biosorption results for dyes using microbial biosorbents in free and/or immobilized form.

Biosorbent	Adsorbate	Biosorption Capacity (mg/g)	Ref.
Calcium alginate beads	Basic black dye	57.70	[45]
*Saccharomyces cerevisiae*	Reactive Red 120	23.48	[46]
*Saccharomyces cerevisiae*	Acid Red 14	91.55	[47]
*Saccharomyces cerevisiae* immobilized in sodium alginate	Brilliant Red HE-3B,	104.67	[20]
*Saccharomyces cerevisiae* immobilized in sodium alginate	Orange 16	410.33	[48]
*Saccharomyces pastorianus* microencapsulated in sodium alginate	Brilliant Red HE-3B	555.55	[8]
*Pseudomonas aeruginosa* immobilized in sodium alginate	Reactive Green 6	21.2	[49]
*Diaporthe schini*	Cristal violet	642.3	[50]
*Penicillium sp.* immobilised in sodium alginate	C.I. Reactive Red	120.48	[51]
*Debaryomyces hansenii* * F39A*	Reactive Blue 19 and Reactive Red 141	7.51 8.16	[52]
*Saccharomyces pastorianus* immobilized in sodium alginate	Brilliant Red HE-3B	224.47	this study

**Table 3:** Thermodynamic parameters for biosorption process of reactive Brilliant Red HE-3B dye onto biosorbent based on residual biomass of *Saccharomyces pastorianus* immobilized in sodium alginate (particle with ϕ 1 = 2 mm).

T (K)	K_L_, L/g	K_L_, L/mol	ΔG^0^ (kJ/mol) $\Delta G=-RT\ln K_{L}$ [41]	ΔH^0^ (kJ/mol)	ΔS^0^ (J/mol K)	χ^2^	RMSE
				$\ln\;K_{L}=\;\;-\;\;\frac{{\Delta H}^{0}}{\mathrm{RT}}+\frac{{\Delta S}^{0}}{R}$ [41]			
278	0.0083 ± 0.002	12.143 ± 2.93	−5.78 ± 0.562	16.96 ± 1.846	79.96 ± 6.190	0.0017	0.0410
303	0.0138 ± 0.001	20.131 ± 1.95	−7.56 ± 0.564				
318	0.0207 ± 2.32 × 10^−5^	30.313 ± 0.03	−9.02 ± 2.93 × 10^−3^				

ΔG, free energy (kJ/mol); ΔH, enthalpy (kJ/mol); ΔS, biosorption entropy changes (kJ/mol K); R, universal gas constant (8.314 J/mol K); T, absolute temperature of solution (K); K_L_, the value of Langmuir constant (L/mol).

**Table 4:** Kinetic parameters for the Brilliant Red HE-3B reactive dye biosorption onto biosorbent based on residual biomass of *Saccharomyces pastorianus* immobilized in sodium alginate (particle with ϕ 2 = 4 mm).

Kinetic Model	Parameter Values
q_exp_ (mg/g) = 11.96	
**Pseudo first order kinetic (Lagergreen model), **$\frac{dq_{t}}{dt}=k_{1}(q-q_{t})$$/\mathrm{lg}(q-q_{t})=\mathrm{lg}q-\frac{k_{1}}{2.303}t$ [57]	
k_1_ (l/min)	0.0057 ± 7.17 × 10⁻^5^
q_e_ (mg/g)	11.0034 ± 0.671
R^2^	0.9926
χ^2^	0.0212
RMSE	0.0486
**Pseudo second order kinetic (Ho model), **$\frac{dq_{t}}{dt}=k_{2}{\left(q-q_{t}\right)}^{2}$$/\frac{t}{q}=\frac{1}{k_{2}q^{2}}+\frac{1}{q}t$ [57]	
k_2_ (g/mg.min)	199.27 ± 1.02 × 10⁻^4^
q_e_ (mg/g)	11.962 ± 1.92 × 10⁻^7^
R^2^	1
χ^2^	3.89273 × 10⁻^8^
RMSE	6.23918 × 10⁻^5^
**Elovich model, **$q_{t}=\frac{\ln(\alpha \;\cdot \;\beta )}{\beta }+\frac{1}{\beta }\ln t$ [57]	
α (mg/g.min)	119.0177
β (g/mg)	2.6288
R^2^	0.9677
**Intra-particle diffusion (Webber–Morris model)**$,\;q=k_{d}t^{1/2}+c$ [55,56]	
k_d1_ (mg/g.min^0.5^)	0.532 ± 0.025
C_1_	0.491 ± 0.286
R^2^	0.9868
χ^2^	0.1044
RMSE	0.1319
k_d2_ (mg/g.min^0.5^)	0.0391 ± 0.020
C_2_	10.553 ± 0.556
R^2^	0.9302
χ^2^	0.0099
RMSE	0.0705
**Film diffusion model (McKay model), **$\ln\;(1-F){=k}_{f}t$ [55,56]	
k_f_ (min^−1^)	0.0057
R^2^	0.9926

k_1_, rate constant of pseudo-first order model, (1/min); q_t_ and q (mg/g), the amounts of dye adsorbed at time *t* and at equilibrium (24 h), respectively; k_2_, rate constant of pseudo-second order model, g/mg min; k_2_q^2^ = h, initial biosorption rate, mg/g min; α, constant about the initial biosorption rate (mg/ g min); β, constant refers to the extent of surface coverage and activation energy for chemisorption (g/mg); k_d_, rate constant for intraparticle diffusion, mg/g min^1/2^; c, intercept to the y axis; k_f_, the rate constant for film diffusion, min^−1^; F, the fractional attainment.
